# Spironolactone for People Age 70 Years and Older With Osteoarthritic Knee Pain: A Proof‐of‐Concept Trial

**DOI:** 10.1002/acr.22724

**Published:** 2016-04-27

**Authors:** Marion E. T. Mcmurdo, Deepa Sumukadas, Peter T. Donnan, Vera Cvoro, Petra Rauchhaus, Ishbel Argo, Helen Waldie, Roberta Littleford, Allan D. Struthers, Miles D. Witham

**Affiliations:** ^1^ University of DundeeDundeeUK; ^2^ Victoria HospitalKirkcaldyFifeUK; ^3^ Ninewells Hospital and Medical SchoolDundeeUK

## Abstract

**Objective:**

To determine whether spironolactone could benefit older people with osteoarthritis (OA), based on a previous study showing that spironolactone improved quality of life.

**Methods:**

This parallel‐group, randomized, placebo‐controlled, double‐blind trial randomized community‐dwelling people ages ≥70 years with symptomatic knee OA to 12 weeks of 25 mg daily oral spironolactone or matching placebo. The primary outcome was between‐group difference in change in Western Ontario and McMaster Universities Osteoarthritis Index (WOMAC) pain subscale scores. Secondary outcomes included WOMAC stiffness and physical function subscores, EuroQol 5‐domain (EQ‐5D) 3L score, and mechanistic markers. Analysis was by intent to treat, using mixed‐model regression, adjusting for baseline values of test variables.

**Results:**

A total of 421 people had eligibility assessed, and 86 were randomized. Mean ± SD age was 77 ± 5 years and 53 of 86 (62%) were women. Adherence to study medication was 99%, and all participants completed the 12‐week assessment. No significant improvement was seen in the WOMAC pain score (adjusted treatment effect 0.5 points [95% confidence interval (95% CI) − 0.3, 1.3]; *P* = 0.19). No improvement was seen in WOMAC stiffness score (0.2 points [95% CI −0.6, 1.1]; *P* = 0.58), WOMAC physical function score (0.0 points [95% CI −0.7, 0.8]; *P* = 0.98), or EQ‐5D 3L score (0.04 points [95% CI −0.04, 0.12]; *P* = 0.34). Cortisol, matrix metalloproteinase 3, and urinary C‐telopeptide of type II collagen were not significantly different between groups. More minor adverse events were noted in the spironolactone group (47 versus 32), but no increase in death or hospitalization was evident.

**Conclusion:**

Spironolactone did not improve symptoms, physical function, or health‐related quality of life in older people with knee OA.

## Introduction

Osteoarthritis (OA) affects over half of the population ages ≥60 years. Management aims to reduce pain, improve function, and improve quality of life, while minimizing the adverse effects of therapy 1. Unfortunately, an appreciable proportion of patients with OA have an inadequate response to existing analgesic treatments 2. Worse still, many existing analgesics, including both opioids and nonsteroidal antiinflammatory drugs (NSAIDs), have frequent and severe side effects, including confusion, constipation, hypertension, fluid retention, worsening of heart failure and renal impairment, and gastrointestinal hemorrhage in the predominantly older population who have OA. In addition, paracetamol, often viewed as the analgesic of choice in later life because of its absence of side effects, has recently been shown to be of questionable effectiveness in osteoarthritic pain 3.

Box 1 **Significance & Innovations**

- Exploration of novel pharmacologic mechanism.
- Target population of older people (mean age 77 years).
- High adherence and completion rates.

Fresh pharmacologic approaches are therefore urgently required for older people with OA to relieve pain and improve quality of life while minimizing adverse effects. We identified spironolactone, a well‐established medication that is an aldosterone antagonist, as a potential novel approach for older people with OA. Spironolactone has a range of antiinflammatory properties 4 of potential relevance to the treatment of OA. There is a wealth of data that aldosterone is proinflammatory, so that by blocking aldosterone, spironolactone may beneficially affect inflammation and pain in OA. In a previous double‐blind trial in 120 functionally impaired people (mean age 75 years) exploring whether spironolactone improved muscle function, 25 mg spironolactone daily was associated with a significant improvement in health‐related quality of life (EuroQol 5‐domain [EQ‐5D] utility score) relative to placebo of 0.10 (95% confidence interval [95% CI] 0.03, 0.18; *P* = 0.006), an improvement that exceeded the minimum clinically important difference for this measure 6. Of the 120 participants, 41% (49 of 120) had self‐reported OA at baseline. Subgroup analysis of the EQ‐5D scores showed that 35% of participants taking spironolactone reported a reduction in pain/discomfort at 20 weeks, compared to only 5% on placebo (*P* < 0.01). We therefore performed a proof‐of‐concept trial of spironolactone in a population of older people with well‐defined knee OA. The trial was designed to provide preliminary evidence about whether spironolactone is more effective than placebo in reducing symptoms of knee pain in older people with OA knee, when given in addition to usual medication.

## Participants and methods

### Design and participants

The study was a randomized, double‐blind, placebo‐controlled, parallel‐group trial. We studied community‐dwelling people ages ≥70 years with knee pain due to OA. Inclusion criteria were as follows: symptomatic idiopathic knee OA according to American College of Rheumatology clinical and radiographic criteria 7, moderate or more severe pain at screening (a score ≥4 on the Western Ontario and McMaster Universities Osteoarthritis Index [WOMAC] pain subscale) in at least 2 of 5 WOMAC pain score items, and receipt of 1 or more analgesic agents at a therapeutic dose for at least 2 months. Exclusion criteria included the following: clinical diagnosis of symptomatic heart failure; history of inflammatory arthritis; already taking spironolactone or previous intolerance; objection to taking capsules made from animal‐sourced gelatin; taking prescribed or over‐the‐counter oral NSAIDs or taking angiotensin‐converting enzyme inhibitors or angiotensin II receptor blockers, because of the potential risk of renal impairment when combined with spironolactone; supine systolic blood pressure (BP) <100 mm Hg at screening; significant chronic kidney disease (estimated glomerular filtration rate [eGFR] <40 ml/minute); serum sodium <130 mmoles/liter; serum potassium >5.0 mmoles/liter; symptomatic orthostatic hypotension at screening; currently receiving a course of physiotherapy; requires a wheelchair; participating in another study; known contraindication to spironolactone therapy; or having a terminal illness.

Participants were recruited from the community via primary care, using the Scottish Primary Care Research Network, and via articles in the local media about the research, previous research participants, and the SHARE National Health Service (NHS) Scotland health research register (www.registerforshare.org). Recruitment took place in 3 Scottish regions (Dundee, Angus, and Fife) between November 2013 and November 2014. All interested potential participants underwent a telephone prescreen, and those who appeared likely to be eligible attended the hospital for an in‐person screen. Research ethics approval was obtained from the West of Scotland Research Ethics Committee (13/WS/0232). Clinical trials authorization was obtained from the UK Medicines and Healthcare Regulatory Authority (European Union Drug Regulating Authorities Clinical Trials No. 2013‐002638‐19). The trial was sponsored by the University of Dundee and NHS Tayside, was registered at clinicaltrials.gov (ISRCTN02046668), and managed by the UK Clinical Research Network registered Tayside Clinical Trials Unit. The protocol is available on request.

### Intervention

Randomization of medication was performed by an independent third party (Tayside Pharmaceuticals) after the baseline assessments had been completed. The randomization code was held by Tayside Pharmaceuticals until after the end of the trial to preserve allocation concealment. After successful screening for eligibility and safety, participants were randomized (1:1 ratio) without blocking or stratification, using sequentially numbered bottles, either to 25 mg spironolactone daily for 12 weeks or to a matching placebo. Participants, health care providers, and researchers were therefore masked to treatment allocation. Participants were allowed to continue all their usual medication throughout.

### Primary and secondary outcome measures

Outcomes were collected at baseline and 12 weeks by 1 of 2 research nurses masked to treatment allocation. The primary outcome was the between‐group difference in change in WOMAC pain subscale (5 items) between baseline and 12 weeks. The WOMAC is a patient‐reported questionnaire 8 that is a valid, reliable, and sensitive instrument recommended for use in clinical studies 9. WOMAC numerical rating scale version 3.1 was administered face‐to‐face 10. Scores were assessed on an 11‐point Likert scale for each subscale (range 0–10, where higher values indicate worsening). Three subscores were created from the questions (pain: 5 questions; stiffness: 2 questions; physical function: 12 questions) as the mean value of all questions for each subscore. The WOMAC stiffness subscale, the WOMAC physical function subscale, and health‐related quality of life measured by EQ‐5D 3L questionnaire were also recorded.

### Other measurements

At 2, 6, and 12 weeks any changes to medication were noted, and blood was taken for sodium, potassium, creatinine, eGFR, and magnesium. The 2‐ and 6‐week blood samples were taken in the participants’ homes. Blood samples were analyzed in the Department of Biochemical Medicine at Ninewells Hospital, Dundee, Scotland.

Serum matrix metalloproteinase 3 (MMP‐3), morning serum cortisol level, and urinary C‐telopeptides of type II collagen (CTX‐II), a marker for type II collagen degradation and osteophyte burden, were measured at baseline and 12 weeks in the Immunoassay Core Laboratory at Dundee Medical School, using enzyme‐linked immunosorbent assay kits with intra‐assay coefficients of 6%, 7.6%, and 12%, respectively. These biomarkers were measured for explanatory purposes with the potential to elucidate the mechanism of spironolactone on symptoms.

### Statistical analysis

The purpose of this trial was proof of concept to acquire preliminary data with which to inform a larger definitive trial. The sample size was 80. The selection of the sample size in such circumstances is, to an extent, arbitrary. In anticipation of a dropout rate of 6.6% at 12 weeks (the dropout rate in our previous trial was 6.6%), we aimed to recruit 86 participants for 80 to complete. This sample size (40 participants randomized to spironolactone, 40 participants randomized to placebo) had 80% power to detect a between‐group difference of 31% in WOMAC pain score. An improvement in WOMAC pain score of 20% is regarded as being of moderate clinical importance, according to the Osteoarthritis Research Society International and Outcome Measures in Rheumatology initiative, and this pilot study clearly would not have enough power to detect this magnitude of difference 8.

Data were analyzed using the SAS 9.2 statistical package in accordance with a prespecified statistical analysis plan. Analyses were performed only after all the data had been entered and the database had been locked. Analyses adjusting for differences in baseline data were performed. Between‐group changes in outcomes were analyzed using intent‐to‐treat analysis. Missing data were handled by multiple imputation, provided that the assumption of missing at random was met. Mixed‐model regression was used to compare differences between the spironolactone and the placebo groups, with adjustment for baseline values.

## Results

**Figure 1:**
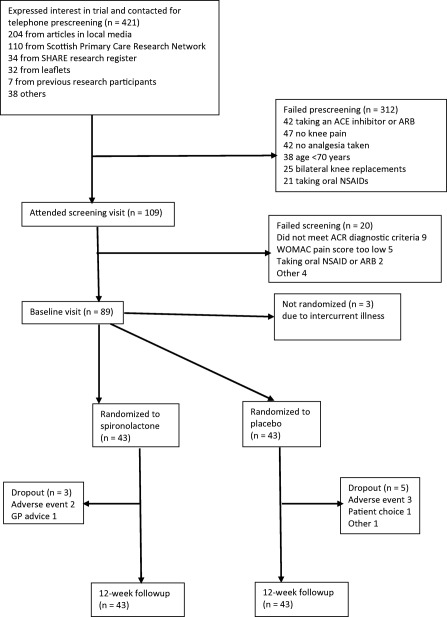
Consolidated Standards of Reporting Trials flow diagram. SHARE = National Health Service Scotland health research register; ACE = angiotensin‐converting enzyme; ARB = angiotensin II receptor blocker; NSAID = nonsteroidal antiinflammatory drug; ACR = American College of Rheumatology; WOMAC = Western Ontario and McMaster Universities Osteoarthritis Index; GP = general practitioner.

A total of 421 participants were assessed for eligibility. Details of the recruitment and followup are given in the Consolidated Standards of Reporting Trials flow diagram (Figure 1). Of the 86 people randomized, 73% (63 of 86) came from articles in the local media and primary care.

The median (range) adherence with medication, derived from tablet counting, was 98% (58–106) in the spironolactone group and 100% (60–100) in the placebo group. The groups were well matched at baseline (Table 1), including for analgesia use.

**Table 1:** Baseline characteristics^a^

	Spironolactone (n = 43)	Placebo (n = 43)
Age, years	77.4 ± 4.8	76.1 ± 5.2
Weight, kg	76.3 ± 15.6	81.3 ± 20.5
Women, no. (%)	26 (60)	27 (63)
Total no. medications	6.9 ± 3.2	6.1 ± 2.8
SIMD 1–5, no. (%)	19 (44)	20 (46)
Systolic blood pressure, mm Hg	141 ± 15	148 ± 15
Diastolic blood pressure, mm Hg	79 ± 9	81 ± 9
Analgesic medication, no. (%)		
Nonopioid preparations	29 (67)	28 (65)
Opioids, weak^b^	16 (37)	17 (40)
Opioids, strong^c^	7 (16)	4 10
Drugs for neuropathic pain^d^	5 12	5 12
Other analgesics^e^	7 (16)	9 (21)
WOMAC pain (0–10)	4.5 ± 1.6	5.3 ± 1.7
WOMAC stiffness (0–10)	5.5 ± 2.0	6.2 ± 1.6
WOMAC physical function (0–10)	4.8 ± 1.7	5.2 ± 1.8
EQ‐5D utility	0.68 ± 0.19	0.60 ± 0.28
EQ‐5D VAS	67.9 ± 16.5	70.4 ± 17.0
Changes in biomarkers		
Urine CTX‐II, μg/liter, median (IQR)	2.1 (1.2–4.7)	2.2 (1.2–4.0)
Serum MMP‐3, ng/ml	25 ± 16	25 ± 15
Morning cortisol, ng/ml	74 ± 35	70 ± 29

a Values are mean ± SD, unless indicated otherwise. SIMD = Scottish Index of Multiple Deprivation (1–5 more deprived, 6–10 more affluent); WOMAC = Western Ontario and McMaster Universities Osteoarthritis Index; EQ‐5D = EuroQol 5‐domain; VAS = visual analog scale; CTX‐II = C‐telopeptides of type II collagen; IQR = interquartile range; MMP‐3 = matrix metalloproteinase 3.

b Codeine, dihydrocodeine.

c Oxycodone, buprenorphine, tramadol.

d Amitriptyline, pregabalin, gabapentin.

e Paracetamol, topical nonsteroidal antiinflammatory drugs.

Medication was withdrawn from 8 participants, 5 in the spironolactone group and 3 in the placebo group. The reasons for withdrawal (n = 1 for each) in the spironolactone group were rash and elevated creatinine level, elevated creatinine level with low potassium, worsening pain that initiated an oral NSAID, and personal reasons. Reasons for withdrawal in the placebo group were elevated creatinine level, delirium, and an initiated course of physiotherapy for knee pain. All 8 continued with study visits as per intent to treat.

### Primary and secondary outcomes

There was no between‐group difference in change in WOMAC pain scores (Table 2). Adjusted analyses also showed no significant difference in change in WOMAC pain score between groups. The amount of missing data on the WOMAC pain score was low at 1.3% (11 of 860 items). Sensitivity analysis using worst‐case values and multiple imputation for missing values did not alter the conclusions. There were no significant between‐group differences in changes in either unadjusted or adjusted scores of WOMAC stiffness and physical function, EQ‐5D 3L utility, and EQ‐5D 3L visual analog scale (VAS) scores (Table 2).

**Table 2:** Unadjusted and adjusted changes at 12 weeks in outcomes and biomarker values by group^a^

	Spironolactone	Placebo	Mixed models^c^			
	(n = 43)^b^	(n = 43)^b^	Unadjusted	*P*	Adjusted^d^	*P*
Primary outcomes						
WOMAC pain^e^	−1.0 (−1.6, −0.4)	−1.7 (−2.3, 1.2)	−0.01 (−0.90, 0.88)	0.9	0.53 (−0.27, 1.33)	0.19
WOMAC stiffness^e^	−1.0 (−1.6, −0.3)	−1.4 (−2.06, −0.73)	−0.20 (−1.18, 0.78)	0.69	0.24 (−0.64, 1.13)	0.58
WOMAC physical function^e^	−1.0 (−1.5, −0.5)	−1.1 (−1.73, −0.50)	−0.30 (−1.19, 0.60)	0.51	0.01 (−0.74, 0.76)	0.98
EQ‐5D^f^	0.02 (−0.05, 0.09)	0.03 (−0.03, 0.10)	0.07 (−0.02, 0.16)	0.13	0.04 (−0.042, 0.12)	0.34
EQ‐5D VAS^f^	0.5 (−4.1, 5.0)	0.14 (−5.23, 5.51)	−2.19 (−9.11, 4.73)	0.53	−0.86 (−6.90, 5.19)	0.78
Changes in biomarkers						
Urine CTX‐II, μg/liter	0.1 (−0.8, 0.9)	−0.03 (−0.81, 0.75)	0.24 (−0.93, 1.41)	0.68	0.21 (−0.83, 1.24)	0.69
Serum MMP‐3, ng/ml	1 (−2, 5)	2.86 (−1.34, 7.07)	−1.44 (−9.00, 6.11)	0.70	−1.98 (−7.32, 3.36)	0.46
Morning cortisol, ng/ml	13 (−1, 27)	8.85 (−2.56, 20.26)	8.15 (−9.62, 25.95)	0.36	6.04 (−10.66, 22.73)	0.47

a Values are mean (95% confidence interval), unless indicated otherwise. WOMAC = Western Ontario and McMaster Universities Osteoarthritis Index; EQ‐5D = EuroQol 5‐domain; VAS = visual analog scale; CTX‐II = C‐telopeptides of type II collagen; MMP‐3 = matrix metalloproteinase 3.

b Difference to baseline.

c Difference between groups (spironolactone versus placebo).

d Adjusted for recruitment site (Fife/Dundee) and baseline values. EQ‐5D also adjusted for the presence of neuropathic drugs at baseline.

e Higher score = worse.

f Lower score = worse.

### Other measurement results

Neither urine CTX‐II or serum MMP‐3 changed significantly during the study in either group (Table 2). Mean morning cortisol levels rose more in the spironolactone group than in the placebo group, a finding consistent with the known effects of spironolactone. However, the between‐group difference in change was not significant.

Safety blood samples showed small statistically but not clinically significant increases in potassium (0.1 versus −0.1 mmoles/liter; *P* < 0.001) and creatinine levels (5 versus 1 μmoles/liter; *P* = 0.01) by 12 weeks in the spironolactone group, as would be expected, but no other changes of significance. BP did not alter significantly in the spironolactone group (systolic BP −2 versus −7 mm Hg; *P* = 0.1; diastolic BP −3 versus −4 mm Hg; *P* = 0.5). During the trial, 5 participants began new analgesic medication, 3 in the spironolactone group (n = 1 weak opioid, n = 1 strong opioid, and n = 1 other analgesic) and 2 in the placebo group (n = 2 other analgesic).

### Adverse events and withdrawals

A total of 47 adverse events occurred in the spironolactone group and 32 in the placebo group. There were more gastrointestinal disorders (10 versus 2) and infections (10 versus 6) in the spironolactone group, but more musculoskeletal disorders (14 versus 9) in the placebo group. There were 5 hospitalizations, 3 in the spironolactone group (1 abdominal pain, 1 anal abscess, and 1 fracture of femoral neck) and 2 in the placebo group (1 exacerbation of chronic obstructive pulmonary disease and 1 delirium). There were no deaths.

## Discussion

This double‐blind study found that spironolactone did not improve WOMAC pain score at 12 weeks compared to placebo. This was a proof‐of‐concept study and deliberately not powered to detect the minimum clinically important between‐group change, but was intended to inform the development of a possible future larger trial. However, there was no signal in the WOMAC pain scores to indicate that spironolactone offered an advantage over placebo; indeed, the 95% CI for the treatment effect did not encompass a 20% improvement, denoting a moderate treatment response compared to baseline. The lack of change in the secondary outcomes and the biomarkers (cortisol, MMP‐3, and urinary CTX II) reinforce this negative finding.

A number of possible explanations for this null result merit consideration. Ceiling effects on WOMAC ratings would not explain the lack of treatment effect, as one of the eligibility criteria was the need to score 4 or more on at least 2 of the 5 WOMAC pain questions.

Nor does a between‐group mismatch in analgesia use seem a likely explanation for the result. The use of mild and strong opioids at baseline was similar between the groups. Analgesic medication altered little during the trial, although more people in the spironolactone group began opioid medication. The effect size in our placebo group was 0.53, comparable to the 0.51 reported by Zhang et al 11 in a meta‐analysis of the placebo effect in 193 OA trials.

There are limitations as to the detail with which we were able to phenotype participants’ disease; in particular, we were not able to ascertain the degree of chronic, low‐grade joint inflammation. Certain subgroups of patients with OA might exhibit greater responses to spironolactone, such as those with a higher degree of chronic synovitis or those with higher baseline turnover of collagen matrix 5.

Possibly the 25‐mg dose of spironolactone was insufficient. However this dose was used in our previous trial, which significantly improved quality of life (using the same EQ‐5D 3L tool) 6, and the same dose is used to prolong life in heart failure 12; the 25‐mg dose in heart failure has been shown to both increase cortisol levels 13 and reduce markers of collagen turnover 14. A larger dose would have increased the likelihood of adverse effects, including raised potassium and creatinine levels, and of gynecomastia in men. The 12‐week duration of treatment was selected for practical and pragmatic reasons. A longer duration of therapy may have been necessary, but the lack of any signal of efficacy in the current study does not lend support to this idea. Furthermore, in our original trial a signal of change in EQ‐5D 3L score was evident after 10 weeks of spironolactone, when the between‐group difference in change from baseline was 0.04 (−0.03 to 1.11; *P* = 0.22) 6. The major difference between the 2 study populations was self‐reported physical disability in the former study, and specific selection for symptomatic OA of the knee in this study. Study populations in both trials were similar in terms of baseline age, sex, weight, social deprivation, EQ‐5D utility score, and EQ‐5D VAS score.

The study has a number of strengths. Recruitment strategies were efficient, with the target number of 86 participants randomized in the preplanned target 12‐month period. Adherence was exemplary, the dropout rate was low, and treatment was well tolerated. This study confirms the safety of 25 mg spironolactone daily in this specific older population with OA. The mean age of participants was 77 years, and there was no upper age limit; this lack of a limit contrasts with around 25% of OA‐related trials, which did impose upper age limits 15, a practice that is increasingly untenable. As most older people are multimorbid, the prospect of a safe, established medication with pleiotropic therapeutic effects is an attractive one. Spironolactone improves outcomes in heart failure and has effects on many cell types, independent of its binding to mineralocorticoid receptors. One drug that could safely treat more than one condition would be a major advance in reducing treatment burden. This proof‐of‐concept study found no evidence to support the further evaluation of spironolactone for older people with knee pain due to OA.

## AUTHOR CONTRIBUTIONS

All authors were involved in drafting the article or revising it critically for important intellectual content, and all authors approved the final version to be submitted for publication. Dr. McMurdo had full access to all of the data in the study and takes responsibility for the integrity of the data and the accuracy of the data analysis.

**Study conception and design.** McMurdo, Sumukadas, Donnan, Struthers, Witham.

**Acquisition of data.** Cvoro, Argo, Waldie, Littleford.

**Analysis and interpretation of data.**McMurdo, Sumukadas, Donnan, Cvoro, Rauchhaus, Struthers, Witham.
